# Comparative Assessment of Observed Dentinal Defects Following Root Canal Preparation With TruNatomy, ProTaper Next, and Dia‐X Nickel–Titanium Rotary Systems: An In Vitro Study

**DOI:** 10.1155/ijod/2003578

**Published:** 2026-10-13

**Authors:** Shakhawan Kadir Kadir, Khanda Ali Kareem

**Affiliations:** ^1^ College of Dentistry Hawler Medical University Erbil Kurdistan Region Iraq hmu.edu.iq

**Keywords:** dentin, dentinal microcracks, endodontics, nickel–titanium, root canal preparation

## Abstract

Endodontic treatment relies on effective root canal preparation using nickel–titanium (NiTi) rotary instruments; however, dentinal defects may be observed following mechanical canal preparation. A total of 40 extracted human mandibular premolars with single roots and single straight canals were randomly allocated into four groups (*n* = 10), control (unprepared), TruNatomy (TRN), ProTaper Next (PTN), and Dia‐X. Root canal was prepared using an ENDO‐MATE DT endodontic motor (NSK, Japan), and horizontally sectioned from the apex using a low‐speed diamond disc, corresponding to apical, middle, and coronal root thirds, respectively. Dentinal microcracks were evaluated under a stereomicroscope at ×20 magnification. Dentinal defects were observed in all instrumented groups, while the unprepared control group showed the lowest incidence, with defects detected at the apical and coronal thirds (10% each). No statistically significant differences were found among the three rotary systems at the apical (*p* = 0.211), middle (*p* = 0.069), or coronal (*p* = 0.600) levels. However, overall comparison showed a statistically significant difference among groups (*p* = 0.043), with significant differences between the control group and TRN (*p* = 0.045), PTN (*p* = 0.045), and Dia‐X (*p* = 0.010). Dentinal defects were detected at different root levels following preparation with all three rotary systems, with higher overall defect incidence than in the unprepared control group; however, the present methodology does not allow definitive attribution of these defects to instrumentation.

## 1. Introduction

Endodontic treatment mainly depends on effective biomechanical preparation of the root canal system to eliminate microorganisms and infected tissues while preserving the structural integrity of the tooth to prevent future reinfection of the sealed root canal system [1, 2]. Biomechanical disinfection is fundamental in endodontic therapy, shaping of the root canal system with different endodontic instruments facilitates cleaning by directly removing bacteria and enhancing antibacterial penetration [3]. However, biomechanical preparation may contribute to microcrack formation due to mechanical stresses generated during root canal shaping [4, 5]. Variations in tooth anatomy, accessibility, and instrumentation techniques may further influence the incidence of dentinal microcrack formation [6]. Microcrack formation may propagate into vertical root fractures (VRFs) and lead to endodontic treatment failure [7]. The introduction of rotary instruments has completely transformed root canal preparation, even though hand instrumentation has long been the basis of endodontic practice [5]. Recently, rotary instruments, especially nickel–titanium (NiTi), have transformed root canal preparation through advantageous instrument design and alloy technology due to their favorable mechanical properties, including superelasticity and shape memory [8–11]. The most commonly used rotary instrument systems are Dia‐X, ProTaper Next (PTN), and TruNatomy (TRN) due to their different structural and functional properties [12, 13]. Compared to conventional rotary systems, TRN offers easier shaping, enhanced safety, greater cutting efficiency, and improved mechanical performance; it is made from patented heat‐treated NiTi wire with an appropriate compact design and reduced taper [14]. With its changeable regressive taper, off‐centered rectangular cross‐section, and offset rotation that lessens canal wall contact and instrument stress, PTN is an M‐Wire NiTi system with enhanced flexibility and cyclic fatigue resistance [15]. Dia‐X files are heat‐treated NiTi instruments with a convex triangular cross‐section, progressive decreasing taper, and noncutting tip, providing improved flexibility, safety, and reduced rotational friction, although limited evidence exists regarding their effect on dentinal integrity and microcrack formation [13, 16].

The present study aimed to comparatively evaluate the incidence of dentinal defects observed following root canal preparation with TRN, PTN, and Dia‐X NiTi rotary systems.

## 2. Materials and Methods

### 2.1. Study Design

An in vitro comparative study was conducted in Erbil city from October 2022 to February 2023 to evaluate the incidence of dentinal defects observed following root canal preparation with three NiTi rotary file systems: TRN, PTN, and Dia‐X. This study addressed 40 extracted human mandibular premolars with single roots and single straight canals. The samples were randomly allocated into three experimental groups (*n* = 10 each) and one control group (*n* = 10). A formal a priori sample‐size calculation was not performed. The sample size was selected based on comparable in vitro studies; therefore, insufficient statistical power for some comparisons cannot be excluded.

Extracted human mandibular premolars with a single straight canal and mature apices were included. Teeth with caries, resorption, clinically evident canal curvature, open apices, previous endodontic treatment, or visible external dentinal defects detected by stereomicroscopic examination were excluded. Before instrumentation, the external root surfaces of all specimens were examined under a stereomicroscope at × 20 magnification to identify visibly detectable dentinal defects. Because this method cannot reliably detect internal pre‐existing microcracks, the absence of defects during initial screening was not considered evidence that the specimens were completely free of internal defects. Digital radiographs (FONA XDG, Italy) confirmed single canals and excluded anatomical abnormalities.

### 2.2. Sample Preparation

The external root surfaces were cleaned using an ultrasonic scaler to remove soft tissue remnants and hard deposits, before being used, teeth were kept at room temperature in distilled water [17]. Using a low‐speed diamond disc under water cooling, all crowns were sectioned at the cemento–enamel junction for standardization. This resulted in a root length of 12 mm, which was verified using a digital caliper (Figure 1). An ISO size 10 stainless‐steel K‐file (0.10 mm tip diameter) was used to establish canal patency. The working length was calculated by advancing the file until its tip was visible at the apical foramen and subtracting 1 mm [18]. A glide path was then created using an ISO size 15 K‐file (0.15 mm), while teeth with apical diameters exceeding size 15 were excluded to standardize canal dimensions [19].

**Figure 1:**
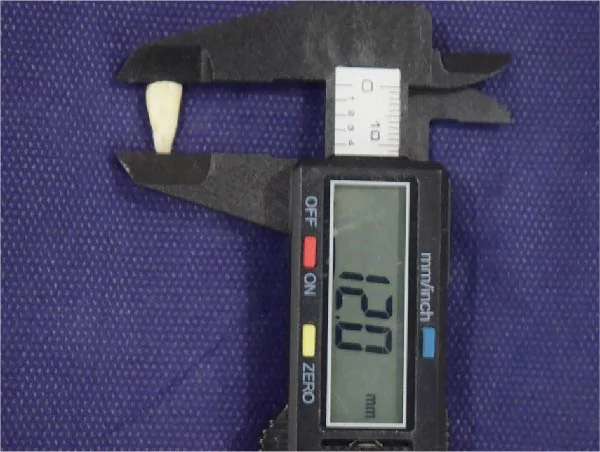
Sample preparation and standardization of root length, canal patency, and glide path establishment.

### 2.3. Periodontal Ligament Simulation and Tooth Mounting

Root surfaces were coated with aluminum foil and embedded centrally in acrylic resin blocks within polyvinyl chloride (PVC) molds, leaving 2–3 mm of the coronal root exposed (Figure 2). After polymerization, the specimens were removed, and the foil was replaced with light‐body silicone material (alphasil perfect, Germany) to simulate the periodontal ligament space [19, 20]. The teeth were then repositioned into the same molds. The specimens (*n* = 40) were randomly divided into four groups (*n* = 10 each), as shown in Table 1.

**Figure 2:**
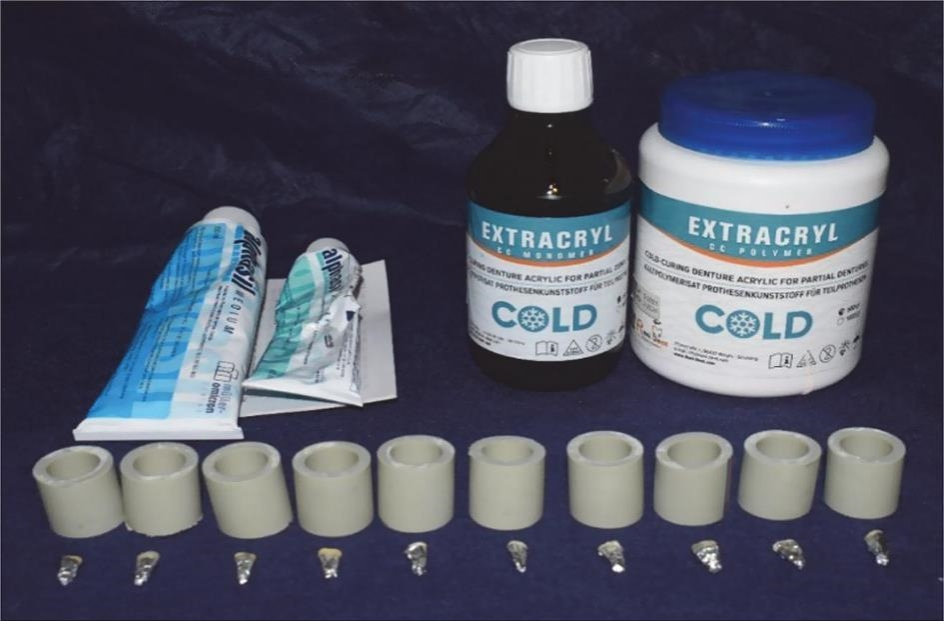
Shows the PVC water pipes prepared to be filled with the acrylic resin and the root samples been covered with aluminum foil embedded in the center of the acrylic resin.

**Table 1:** Experimental grouping of specimens according to root canal instrumentation systems.

Group	Description
Group 1	Control (unprepared canals)
Group 2	Root canal preparation using TruNatomy
Group 3	Root canal preparation using ProTaper Next
Group 4	Root canal preparation using Dia‐X

### 2.4. Root Canal Instrumentation Protocol

Mechanical preparation was carried out using an ENDO‐MATE DT endodontic rotary motor (NSK, Japan). Before instrumentation, 1 mL of 5.25% sodium hypochlorite (NaOCl) was introduced into the canal to avoid dry instrumentation. In the TRN group, canals were prepared using TRN Glider (17/.02), followed by TRN Prime (26/.04) at 500 rpm and 1.5 Ncm torque. In the PTN group, instrumentation was performed using X1 (17/.04) and X2 (25/.06) files at 300 rpm and a 2 Ncm torque. In the Dia‐X group, D2 (17/.04) and D4 (25/.07) files were used at 300 rpm and 2.4 Ncm torque.

Instrumentation was performed using a crown‐down technique with continuous rotary motion and gentle in‐and‐out pecking movements until the working length was reached. After each instrument, canals were irrigated with 2 mL of 5.25% NaOCl delivered using a 30‐gauge side‐vented irrigation needle positioned 2 mm short of the working length. Canal patency was maintained using a size #15 K‐file. Five milliliters of regular saline were used for the final irrigation, and absorbent paper points were used to dry the canals [21].

### 2.5. Sectioning and Dentinal Microcrack Evaluation

After instrumentation, a low‐speed diamond disc under continuous water cooling was used to horizontally section the roots at 3, 6, and 9 mm from the apex, corresponding to the apical, middle, and coronal root thirds, respectively. Because the sectioning procedure was destructive, the possibility that the sectioning process could create or propagate dentinal defects cannot be completely excluded. The samples were fixed on the wax blocks after cutting for handling and standardization, as shown in Figure 3. The sections were examined for dentinal defects under a stereomicroscope at ×20 magnification [22]. Dentinal defects were classified according to the criteria described by Karataş et al. [23] as either present or absent; defects classified as present included craze lines, incomplete cracks, or complete cracks. The present study did not perform a separate assessment of defect severity, length, depth, or extent. Interexaminer agreement was assessed using the weighted kappa statistic. Very good agreement was observed for the middle (*κ* = 0.862, *p*  < 0.001) and coronal (*κ* = 0.760, *p*  < 0.001) regions. No kappa value was calculated for the apical region because complete agreement was observed between the examiners.

**Figure 3:**
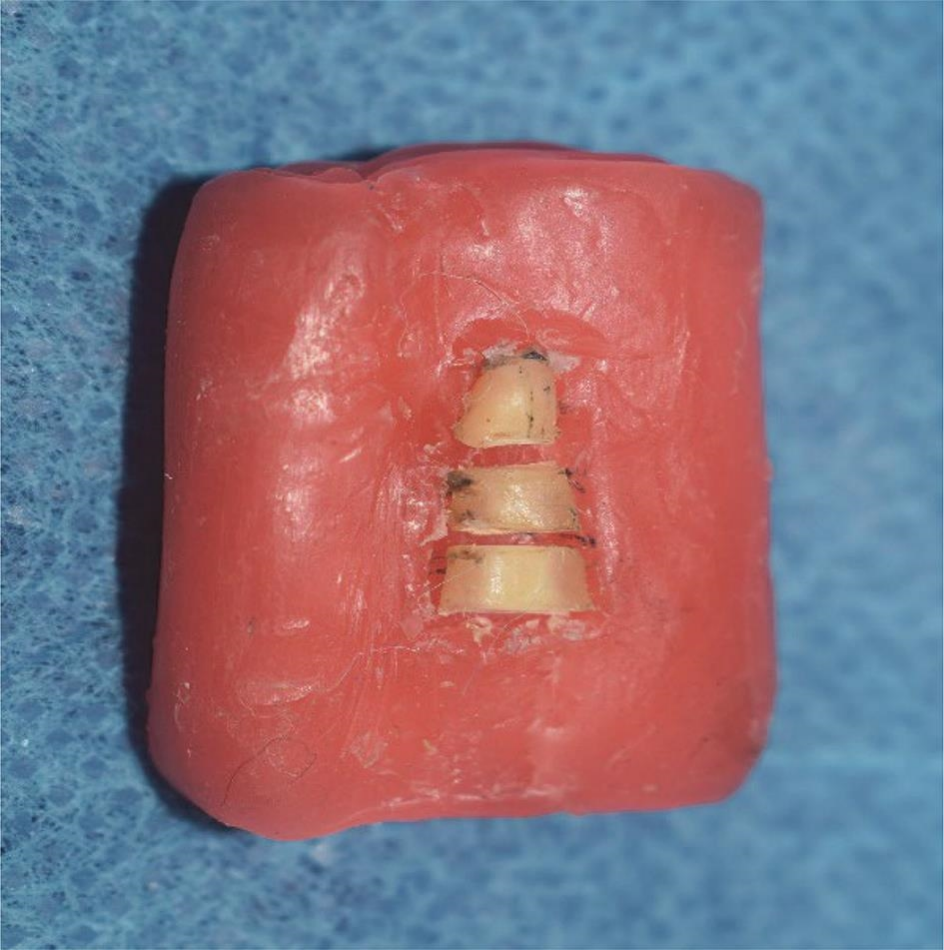
Post sectioning fixed in a wax block.

### 2.6. Statistical Analysis

Statistical analyses were performed using SPSS Version 26.0 (IBM Corp., Armonk, NY, USA). Descriptive data were presented as frequencies and percentages. Because the primary outcome was categorical (defect present/absent), comparisons of dentinal defect incidence among groups were performed using the chi‐square test. The assumptions underlying the categorical‐data analysis were assessed before analysis. Normality and homogeneity of variance were not applicable to the primary categorical outcome. Statistical significance was set at *p*  < 0.05.

## 3. Results

### 3.1. Observed Dentinal Defects at Different Root Levels

The distribution and percentage of observed dentinal defects at different root levels are presented in Table 2 and Figure 4. The control group showed the lowest incidence of dentinal defects, with only one defect observed at the apical and coronal levels (10%) and no defects detected at the middle level. Representative stereomicroscopic images of intact (nondefected) specimens are shown in Figure 5. Among the instrumented groups, the apical region showed the highest observed incidence of dentinal defects across the three rotary systems. TRN exhibited the highest percentage of defects in the apical third (50%), followed by both PTN and Dia‐X (40% each). In the middle third, Dia‐X showed the highest defect incidence (40%), while TRN and PTN demonstrated 30% each. The coronal third showed the lowest percentage of defects across all groups. Representative images of dentinal defects are illustrated in Figure 6, where microcracks are indicated by black arrows.

**Figure 4:**
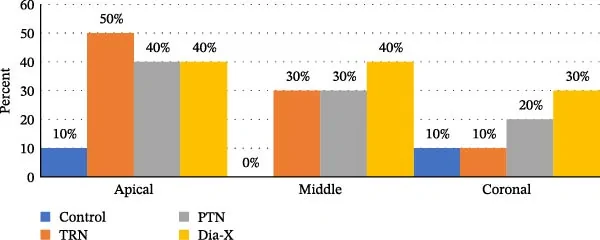
Percentage distribution of dentinal microcracks at apical, middle, and coronal root levels among the study groups.

**Figure 5:**
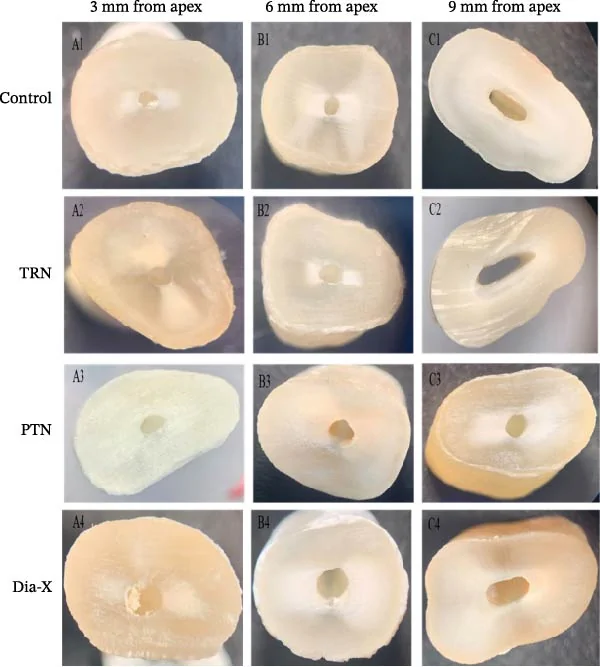
Shows images of not defected samples taken with stereomicroscope at 20x magnification at three root sections for four groups, control (A1, B1, C1), TRN (A2, B2, C2), PTN (A3, B3, C3), and Dia‐X (A4, B4, C4).

**Figure 6:**
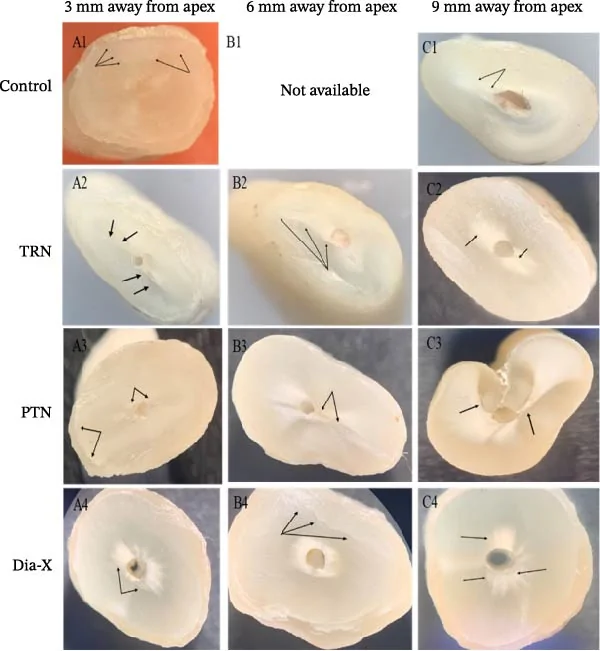
Representative stereomicroscopic images of dentinal defects detected at ×20 magnification in the control, TruNatomy, ProTaper Next, and Dia‐X groups. Black arrows indicate the observed defects.

**Table 2:** Distribution of dentinal microcrack formation at different root levels.

Groups	Status	Apical, *n* (%)	Middle, *n* (%)	Coronal, *n* (%)	Total
Control	Defect	1 (10)	0 (0)	1 (10)	2 (7)
TRN	No defect	9 (90)	10 (100)	9 (90)	28 (93)
	Defect	5 (50)	3 (30)	1 (10)	9 (30)
PTN	No defect	5 (50)	7 (70)	9 (90)	21 (70)
	Defect	4 (40)	3 (30)	2 (20)	9 (30)
Dia‐X	No defect	6 (60)	6 (60)	7 (70)	19 (63)
	Defect	4 (40)	4 (40)	3 (30)	11 (37)

### 3.2. Between‐Group Comparison of Dentinal Microcrack Formation

The chi‐square test showed no significant differences between groups at the apical (*p* = 0.211), middle (*p* = 0.069), or coronal (*p* = 0.600) levels. However, a significant overall difference was observed when all root levels were combined (*p* = 0.043), Table 3. Significant differences were observed between the control group and TRN (*p* = 0.045), PTN (*p* = 0.045), and Dia‐X (*p* = 0.010), while no significant differences were detected among the three rotary systems (Table 4).

**Table 3:** Comparison of dentinal microcrack formation among groups at different root levels.

Root level	Chi‐square value	*p*‐Value
Apical	4.511	0.211
Middle	7.092	0.069
Coronal	1.869	0.600
Overall	8.133	0.043 ^∗^

^∗^Indicates statistical significant at *p* < 0.05.

**Table 4:** Pairwise comparison of overall dentinal microcrack formation between groups.

Groups	Comparison	*p*‐Value
Control	TRN	0.045 ^∗^
	PTN	0.045 ^∗^
	Dia‐X	0.010 ^∗^
Dia‐X	TRN	0.584
	PTN	0.584
PTN	TRN	1.000

^∗^Indicates statistical significant at *p* < 0.05.

### 3.3. Within‐Group Comparison Among Root Levels

Within‐group comparison between root levels showed no statistically significant differences in dentinal microcrack formation between the apical, middle, and coronal regions in any study group (*p*  > 0.05) (Table 5).

**Table 5:** Within‐group comparison of dentinal microcrack formation among root levels.

Groups	Pairwise comparison	*p*‐Value
Control	Apical vs. middle	1.000
	Apical vs. coronal	1.000
	Middle vs. coronal	1.000
TRN	Apical vs. middle	0.650
	Apical vs. coronal	0.141
	Middle vs. coronal	0.582
PTN	Apical vs. middle	1.000
	Apical vs. coronal	0.628
	Middle vs. coronal	1.000
Dia‐X	Apical vs. middle	1.000
	Apical vs. coronal	1.000
	Middle vs. coronal	1.000

## 4. Discussion

Biomechanical preparation is essential for eliminating microorganisms, removing debris, and shaping the root canal to facilitate adequate obturation [3, 24]. However, contact between endodontic instruments and canal walls may generate dentinal stress, resulting in craze lines and microcracks that can propagate into VRFs and compromise tooth survival over time [25–27]. NiTi rotary devices are widely used in modern endodontics due to their efficiency, quicker instrumentation times, and improved canal shaping with fewer procedural mistakes [28, 29]. In the present study, extracted mandibular premolars were selected, and stereomicroscopic examination at × 20 magnification was performed prior to instrumentation to exclude visibly detectable external dentinal defects. To simulate periodontal support, roots were coated with silicone impression material to mimic the periodontal ligament, although this material may not completely reproduce the viscoelastic properties of the natural ligament [19, 30, 31].

The destructive sectioning approach used in the present study represents an important methodological limitation. Versiani et al. [32] critically highlighted the methodological limitations of sectioning‐based approaches for dentinal microcrack research, including the possibility that sectioning may introduce or propagate dentinal defects. Therefore, the defects detected in the present study cannot be definitively attributed to the instrumentation. NiTi engine‐driven instruments are now essential in root canal biomechanical preparation, offering reduced treatment time, fewer procedural errors, improved canal cleanliness, and decreased operator fatigue [28].

In the present study, instrumentation was performed using gentle in‐and‐out pecking movements without an intentional brushing motion, and therefore, the potential effect of an intentional brushing motion was not evaluated. Recent micro‐CT studies have reported that brushing motion does not necessarily increase dentinal microcrack formation; however, differences in instrumentation motion should be considered when comparing findings across studies [33].

Dentinal defects were observed in all three rotary instrumentation groups in the present study, which is consistent with previous studies reporting dentinal defects following root canal preparation with rotary instruments [34, 35]. This may be attributed to the increased rotational stresses generated during canal preparation. In contrast to previous studies reporting defect‐free control groups [36, 37], isolated dentinal defects were also observed in the unprepared control group in the present study, with one specimen showing a defect in the apical third (10%) and one specimen showing a defect in the coronal third (10%). The presence of defects in uninstrumented specimens may reflect pre‐existing defects that were not detectable during initial stereomicroscopic screening or defects associated with extraction, storage, handling, or subsequent sectioning procedures.

Stereomicroscopic examination during tooth selection permits detection of visibly detectable external root surface defects but may fail to identify pre‐existing internal dentinal microcracks before instrumentation or sectioning [38]. Therefore, the absence of defects during initial stereomicroscopic screening cannot be considered evidence that all specimens were free of internal pre‐existing microcracks. This limitation was supported by micro‐CT studies demonstrating the presence of undetected pre‐existing microcracks in nonendodontically treated teeth, despite the absence of defects under stereomicroscopic evaluation [39, 40]. The detection of dentinal defects may also be influenced by methodological factors, including the use of extracted teeth, extraction‐related stresses, storage conditions, irrigation protocols, handling, and the destructive sectioning procedure [18, 41]. Consequently, the defects observed in the present study cannot be definitively classified as newly developed defects resulting from the instrumentation. High‐concentration sodium hypochlorite may alter dentin mechanical properties and could potentially influence the detection or development of dentinal defects [42, 43]. In the present study, the apical region showed a higher observed incidence of defects in the TRN (50%), PTN (40%), and Dia‐X (40%) groups than in the middle and coronal regions. This distribution may be associated with the confined anatomy of the apical region and the mechanical stresses occurring during instrumentation [18, 28, 44]. However, because the present methodology did not permit the direct identification of newly formed defects, this possible association should be interpreted cautiously. The limited anatomy and smaller canal diameter at this level may increase mechanical stress and apical pressure during instrumentation and could partly explain the higher observed incidence of dentinal defects in the apical third [44]. However, the present study cannot establish that these stresses directly caused the observed defects. Furthermore, dentin removal during canal shaping may weaken the root structure, increasing the risk of vertical fracture and making the apical region more prone to defect formation, especially in teeth with less dentin remaining [45, 46]. Dentinal defects were detected at the apical, middle, and coronal root levels in the instrumented groups, and the overall incidence of detected defects differed significantly between the instrumented groups and the untreated control group (*p*  = 0.045 and 0.010, *p*  < 0.05). Nevertheless, the significant differences between groups should be interpreted as differences in the incidence of defects detected after instrumentation rather than definitive evidence of newly formed instrument‐induced microcracks.

These findings are consistent with previous studies reporting an association between mechanical preparation and dentinal defect detection using stereomicroscopy and root sectioning methods [47], although micro‐CT‐based research has questioned whether these defects represent newly formed microcracks [48]. Additionally, differences in observed dentinal defect incidence may potentially be related to instrument design features such as taper, cross‐sectional geometry, tip design, pitch, and alloy treatment [49, 50].

Although the observed percentages differed numerically among the groups, the difference in coronal defect incidence was not statistically significant (*p* = 0.600). Therefore, no statistically supported difference in coronal defect incidence among the three rotary systems can be established. Its regressive taper, off‐centered parallelogram cross‐section, and thin heat‐treated NiTi wire, which may reduce canal wall contact during instrumentation, could potentially contribute to the lower observed incidence of coronal defects in the present study [51]. The extra use of the TRN orifice modification may have further helped to improve canal preparation and lessen coronal stress [52]. TRN had more apical defects (5/10) than PTN and Dia‐X (4/10 each), with its conservative design, possibly due to its larger apical tip size (26 vs. 25) and the use of fewer shaping instruments, which may increase localized stress on canal walls [53]. In contrast, previous studies reported fewer apical defects with TRN, potentially due to differences in the instrumentation sequence and irrigant concentration [54]. Dia‐X demonstrated a greater observed incidence of defects at the middle and coronal thirds, which may potentially be related to its convex triangular cross‐section and 7% taper and their effects on dentin contact and stress transmission during instrumentation. PTN produced more defects than TRN, consistent with previous studies, possibly due to its off‐centered rectangular cross‐section and variable taper, which generate a swaggering motion and a wider cutting envelope that may increase dentinal stress [19, 37]. Additionally, differences in rotational speed and torque among the tested systems (TRN 500 rpm/1.5 N · cm, PTN 300 rpm/2 N · cm, and Dia‐X 300 rpm/2.4 N · cm) may have influenced the observed incidence of dentinal defects, as higher torque settings have previously been associated with greater dentinal stress and crack formation [55, 56].

## 5. Conclusions

Within the limitations of the present in vitro methodology, dentinal defects were observed at the apical, middle, and coronal root levels following root canal preparation with TRN, PTN, and Dia‐X NiTi rotary systems. The apical third demonstrated the highest observed incidence of dentinal defects in the instrumented groups, with TRN showing 50% and PTN and Dia‐X each showing 40%. Dia‐X demonstrated the highest observed defect incidence at the middle (40%) and coronal (30%) levels, whereas, TRN showed a numerically lower observed coronal defect incidence; however, this difference was not statistically significant and therefore should not be interpreted as evidence of superiority. However, because initial screening was performed using stereomicroscopy and final assessment required destructive root sectioning, the present study cannot definitively determine whether the detected defects were newly developed during instrumentation or were pre‐existing or procedure‐related; therefore, the findings should be interpreted as observed dentinal defect incidence rather than definitive evidence of instrument‐induced microcrack formation.

## 6. Limitations

Several methodological limitations should be considered when interpreting the present findings. First, dentinal defects were assessed using stereomicroscopy after destructive root sectioning rather than nondestructive pre and postinstrumentation imaging. Therefore, the method cannot reliably identify internal pre‐existing defects or establish whether an observed defect developed during instrumentation. Second, extraction, storage, handling, and sectioning may have contributed to the detection or development of dentinal defects. Third, the defects were recorded dichotomously as present or absent without an assessment of their severity, length, depth, or extent. Fourth, a formal a priori sample‐size calculation was not performed, which may limit the statistical power of some comparisons. Finally, canal curvature was assessed qualitatively rather than using a predefined angular threshold, which may have introduced anatomical variability among the specimens.

**Nomenclature**
CC BY: Creative Commons Attribution license
Dia‐X: Dia‐X nickel–titanium rotary file system
ENDO‐MATE DT: Endodontic motor system (NSK, Japan)
K‐file: Stainless steel hand endodontic file
LD: Linear dichroism
NaOCl: Sodium hypochlorite
NiTi: Nickel–titanium alloy
PTN: ProTaper Next (nickel–titanium rotary system)
PVC: Polyvinyl chloride
SPSS: Statistical Package for the Social Sciences
TLA: Three‐letter acronym
TRN: TruNatomy (nickel–titanium rotary system)
VRF: Vertical root fracture
*n*: Sample size
rpm: Revolutions per minute.

## Author Contributions

Shakhawan Kadir Kadir contributed to supervision, project administration, validation, and reviewed and edited the manuscript. Khanda Ali Kareem performed the conceptualization, methodology, investigation, data collection, analysis, and wrote the original draft.

## Funding

This research received no external funding.

## Disclosure

All authors have read and agreed to the published version of the manuscript.

## Ethics Statement

The study was conducted in accordance with the Declaration of Helsinki, and approved by the Ethics Committee of Hawler Medical University, College of Dentistry.

## Conflicts of Interest

The authors declare no conflicts of interest.

## Data Availability

All the data are available upon request.
